# Artificial neural networks for predicting social comparison effects among female Instagram users

**DOI:** 10.1371/journal.pone.0229354

**Published:** 2020-02-25

**Authors:** Marta R. Jabłońska, Radosław Zajdel

**Affiliations:** Department of Computer Science in Economics, Faculty of Economics and Sociology, Institute of Logistics and Informatics, University of Lodz, Lodz, Poland; Wroclaw University of Science and Technology, POLAND

## Abstract

Systematic exposure to social media causes social comparisons, especially among women who compare their image to others; they are particularly vulnerable to mood decrease, self-objectification, body concerns, and lower perception of themselves. This study first investigates the possible links between life satisfaction, self-esteem, anxiety, depression, and the intensity of Instagram use with a social comparison model. In the study, 974 women age 18–49 who were Instagram users voluntarily participated, completing a questionnaire. The results suggest associations between the analyzed psychological data and social comparison types. Then, artificial neural networks models were implemented to predict the type of such comparison (positive, negative, equal) based on the aforementioned psychological traits. The models were able to properly predict between 71% and 82% of cases. As human behavior analysis has been a subject of study in various fields of science, this paper contributes towards understanding the role of artificial intelligence methods for analyzing behavioral data in psychology.

## Introduction

Social media are significantly changing the way people interact, increasing the role of user-generated content that is also subject to feedback from other users [1,2]. Given that social networking sites (SNSs) provide rich opportunities for social comparison [3], researchers have begun to explore the consequences for psychological well-being [4]. By spending a vast amount of time viewing others’ posts, users are inevitably drawn into the process of social comparison, especially when using SNSs dedicated to visual content, such as Instagram [5]. Social comparison research examines how individuals react when comparing themselves with others. It distinguishes two acts of comparisons: upward and downward. People make an upward comparison when comparing themselves unfavorably to others. By contrasting themselves with those they perceive as somehow better, individuals may display unpleasant and painful emotions, or it may form part of the drive to self-improvement. Downward comparison takes place when people compare themselves favorably to others whom they perceive as worse off. It can help to restore threatened self-esteem, but also happiness and pride [5,6]. Thus, both comparisons are not always straightforward, and they can have a positive or negative effect on the development of self-evaluation and identity [1,6,7].

The effects of regular exposure to social media posts causing social comparisons are an important area to research [8,9]. Women, in particular, compare their image to others, so they are particularly vulnerable to self-objectification, anxiety, body satisfaction, and lower perceptions of themselves [9,10]. In this paper, social comparisons made by young women due to Instagram usage were researched. The links between types of social comparisons, the intensity of using Instagram, and selected psychological traits were studied using the comparison-identification-contrast model [11,12]. Thanks to this model, people make comparisons by identifying or contrasting themselves with others, where ‘identification’ refers to closeness to the target and ‘contrast’ to distance. By differentiating the upward and downward interpretation in each comparison, four possible feelings may arise while making social comparisons: upward-contrast indicates inferiority, downward-contrast–superiority, upward-identification–hope, and downward-identification–disappointment [12]. The results were used to construct a set of artificial neural networks (ANNs) that can be used to diagnose the intensity of each type of social comparison based on derived psychological traits. As predicting behavior is a classical problem of psychology [13], human behavior analysis has become a subject of study in artificial intelligence methods for analyzing behavioral data [14,15]. ANNs are able to analyze data, learn from them, and then make classifications, diagnoses, or predictions [16]. They are often called “black boxes”, tools where a researcher has little concern about what is going on inside. As it may appear as a flaw, it is actually an advantage, because ANNs don’t require physical pre-information before modeling a system, and ANNs are used when there is a need for complex answers and algorithms and possible relations between data are unknown. There is a wide range of functionalities provided by these algorithms what makes them promising for use in predicting in psychology, including: mental health, behavior, emotions, and personality traits [17–21]. ANNs approaches explicitly concentrate on statistical learning of nonlinear functions from multidimensional data sets to make further generalized predictions about data vectors not seen during training. Thus, they have a potential to boost decisions associated with the diagnosis, prognosis, and treatment in psychology and psychiatry [22]. Still, despite various promising studies, the automated understanding of the behavior, emotions or personality traits of both individuals and groups stays a challenging issue to artificial intelligence techniques [23]. As current psychological studies concentrate highly on explaining the roots of behavior using complex psychological mechanism theories with poor predicting abilities, ANNs implementations could help psychology become a more predictive science that can induce better understanding of human behavior [24].

The paper aimed to: 1) study if selected psychological characteristics (self-esteem, life satisfaction, depression, anxiety) and the intensity of Instagram usage are related to social comparisons made by young women; 2) test the possibility of implementing artificial neural networks in psychological data to predict the intensification of social comparisons. Thus, the paper is structured as follows. Section 2 starts with a description of the current online literature on social comparisons that was used to identify the psychological characteristics used in the study. Next, all details concerning the questionnaire survey and the analysis of the resulting data are provided. Finally, it describes the methods used to construct artificial neural networks. Section 3 provides the results of the questionnaire survey as well as the performance of the artificial neural network. The paper ends with a discussion and conclusions presenting the limitations of the study and avenues of future research.

## Materials and methods

### Selection of the psychological traits

To define which characteristics should be included in the study, an analysis of the current literature was performed using CiteSpace software. It is a freely available tool for interactive and exploratory analysis of the evolution of a scientific domain, ranging from a single specialty to multiple interrelated scientific frontiers, and it was instrumental in revealing insightful patterns from the set of relevant publications [25, 26]. The analysis was made based on 305 articles on online social comparisons from the Web of Science database published between 2014 and 2019 (details provided in S1 Dataset: CiteSpace input text file and S2 Appendix: CiteSpace analysis criteria). They were analyzed, clustered and labeled, with each set representing an underlying theme, topic, or line of research. Detailed cluster descriptions extracted from CiteSpace are presented in S1 Table. The final citation network created by CiteSpace comprised five clusters, including 126 papers. Among them 49 were selected by CiteSpace as the most influential in the main areas of online social comparison studies (S1 Appendix). The biggest, first cluster was dedicated mainly to works on body dissatisfaction caused by Instagram usage. It referred also to terms including social comparison, instagrammers’ body image, ideal appearance, thinspiration, fitspiration communities, and young women. The second cluster was defined by terms social comparison and subjective well-being. Among papers classified to this set, following topics were the most popular: social media use impact and motives, personality traits, communication type, envy, and depression. Millennial population and specific social media behaviors described the third cluster, dedicated also to studies on depressive disorder, identity distress, social comparison, and psychological well-being. The fourth cluster emphasized a role of self-comparison and body image dissatisfaction. Finally, the last cluster was consisting of research on internet support group, predictors of anxiety and depression, young women, and anonymous online settings. CiteSpace analysis helped us to find the most influential works on subject of online social comparisons. Manuscripts from the clusters were searched for studies describing possible links between online social comparisons and psychological characteristics. A set of traits that were mentioned repeatedly was selected for further questionnaire study. Among other psychological traits, present in clusters, following can be mentioned: adult attachment, narcissism, envy, pride, self-objectification, loneliness, and happiness. As they appeared rarely, in limited number of papers, we excluded them from our study, concentrating on the most often occurring traits presented in Table 1.

**Table 1:** Works describing psychological traits possibly related to online social comparisons.

Psychological trait	Works suggesting a relation
Anxiety	Powell, 2018 [27]; Alberga, Withnell and von Ranson, 2018 [28]
Depression	Verduyn et al., 2017 [1]; Lup, Trub and Rosenthal, 2015 [4]; Utz and Muscanell, 2018 [6]; Alberga, Withnell and von Ranson, 2018 [28]; Weinstein, 2017 [30]; Turner and Lefevre, 2017 [31]; Chow and Wan, 2017 [33]; Robinson et al., 2019 [37]
Self-esteem	Verduyn et al., 2017 [1]; Thomas et al., 2017 [5]; Alberga, Withnell and von Ranson, 2018 [28]; Yang, Holden and Carter, 2018 [36]; Tiggemann and Zaccardo, 2015 [39]
Life satisfaction	Verduyn et al., 2017 [1]; Utz and Muscanell, 2018 [6]; Weinstein, 2017 [30]; Chae, 2018 [34]; Meier and Schäfer, 2018 [35]
SNSs usage intensity	Lup, Trub and Rosenthal, 2015 [4]; Fardouly, Willburger, Vartanian, 2018 [29]; Gerson, Plagnol and Corr, 2016 [32]; Yang and Robinson, 2018 [38], Walker et al., 2015 [40]; Kim and Chock, 2015 [41]

### Measures

To collect data, a questionnaire survey was conducted (S3 Appendix). The psychological traits described in the previous section (Table 2) were used in the questionnaire. Anxiety and depression were assessed using the 14-item Hospital Anxiety and Depression Scale (HADS) [42]. The HADS is a frequently used self-rating scale, comprised of subscales of anxiety and depression, developed to assess psychological distress in non-psychiatric patients. It is attractive to clinicians and researchers who need a rapid, efficient assessment [43] and has demonstrated satisfactory psychometric properties in assessing the symptom severity and caseness of anxiety disorders and depression in both somatic, psychiatric and primary care patients; in the general population as well as in different groups, including primary care patients, cognitively intact nursing home patients, and cancer inpatients [44–50]. Internal consistency was high in the present sample (Cronbach’s α = 0.81). Self-esteem was measured with the Rosenberg 10-item scale [51], which is widely used in social-science research and measures global self-worth by assessing both positive and negative feelings about the self like: “On the whole, I am satisfied with myself”, “I feel that I have a number of good qualities” or “I feel I do not have much to be proud of”. Positive and negative items were presented alternately in order to reduce the effect of respondent set. While the reader may question one or another item, there is little doubt that the items generally deal with a favorable or unfavorable attitude toward oneself [51]. Internal consistency in the present sample was Cronbach’s α = 0.84. To evaluate life satisfaction, the Satisfaction With Life Scale (SWLS) [52] was used. It has been deployed heavily as a measure of the life satisfaction component of subjective well-being. In the area of health psychology, the SWLS has been used to measure the subjective quality of life of people experiencing serious health concerns [52] The SWLS is a five-item instrument consisting of statements: “In most ways my life is close to my ideal”, “The conditions of my life are excellent”, “I am satisfied with my life”, “So far I have gotten the important things I want in life”, and “If I could live my life over, I would change almost nothing”. It has been shown to correlate with measures of mental health and to be predictive of future behaviors [53]. For the analyzed sample, internal consistency was high (Cronbach’s α = 0.82). To measure the intensity of Instagram usage, the 13-item Facebook Intensity Scale [54] was adopted. This scale is used to measure Facebook usage beyond simple measures of frequency and duration, incorporating emotional connectedness to the site and its integration into individuals’ daily activities; it can separate problematic and non-problematic aspects of Facebook use [55]. Internal consistency in the present sample was Cronbach’s α = 0.79. Finally, a 12-item social comparison scale [56] was used after being reconfigured for Instagram usage. It was developed in order to measure identification and contrast with upward and downward comparison others. An upward comparison occurs when someone compares himself unfavorably to others (i.e. “When I compare my Instagram profile to people who have better profiles, I feel frustrated with the level of my own profile”), while downward comparison takes place when compares favorably to others whom they perceive as worse off (i.e. “When I compare my Instagram profile to people who have weaker profiles, I feel how good I am doing). Internal consistency was high in the present sample (Cronbach’s α = 0.86).

**Table 2:** Aggregation of social comparisons.

Type of social comparison	Results
Positive: upward identification + downward contrast	566
Negative: downward identification + upward contrast	154
Equal	254
Total	974

A total number of questions was 54. In the original versions, the measures used possessed different Likert scales. And so, HADS and Rosenberg self-esteem scale had a 4-point scale, Facebook Intensity Scale used a 5-point scale, while SWLS and social comparison scale were measured with 7-point Likert scale. To make our questionnaire more clear to respondents, all items were modified by implementing a 7-point Likert scale (from strongly agree to strongly disagree). Likert scales are very useful and the most common approach in opinion surveys for several reasons including they are relatively easy to write and respondents are familiar with such questions [57]. The use of Likert scales, is a common means of assessing people’s attitudes, values, internal states, and judgments about their own or others’ behaviors in both research and clinical practice [58]. Respondents are asked to rank their agreement (from strongly disagree to strongly agree) with a set of items on a scale that has a limited number of possible responses that are presented in a sequence for a real or hypothetical situation under study [57]. Further this data set can be statistically treated with Pearsons’ correlation coefficient, ANOVA, and regression analysis [59]. We can distinguish symmetric and asymmetric Likert scales. In the first type, the position of neutrality (i.e. “I don’t know”) lies exactly in between two extremes of strongly disagree to strongly agree, providing independence to a participant to choose any response in a balanced and symmetric way in either directions. The asymmetric Likert scale offer less choices on one side of neutrality than the other side, so it can cause forced choices where there is no perceived value of neutrality of the researcher [59,60]. Likert scale originally consists of five items, but different variations also occur, such as seven or ten point scales. In such cases adjacent options are less radically different from each other as compare to a 5 point scale. It may help participant to pick the most preferable rather than a “nearby” one [61]. A seven point Likert scale would generate data that can be used as interval data with a lower measurement error and a correspondingly higher precision when compared with the five point original scale [62].

Respondents filled in the questionnaire using text values: strongly agree, agree, rather agree, neither agree or disagree, rather disagree, disagree, and strongly disagree. During data preprocessing, these values were changed to numerical ranging from 1 (strongly disagree) to 7 (strongly agree). So the higher score respondent gained for particular measure, the higher level of psychological trait was evinced. Some questions (i.e. in HADS) were reversed. That means that selecting “strongly agree” indicated respondent’s activity or belief decreasing level of a measured trait. The scales of these questions were reversed at the point of data preprocessing to get the calculations correct. The total sum for each trait was an input for a neuron network. That is why, our networks had 5 neurons an input layer, one for each psychological trait (anxiety, depression, self-esteem, life satisfaction, and Instagram usage intensity).

### Participants and data collection

The sample comprised 974 women age 18–49 who were Instagram users. Of the participants, 20.8 percent were aged 18–20, 68.5 percent were in their twenties, 7.6 percent were aged 30–39, and 3.1 percent were 40–49. All participants were Caucasian women, of Polish origin. The respondents voluntarily accessed an online questionnaire through a link advertised on social media (Facebook and Instagram). The study was advertised as an anonymous, confidential questionnaire investigating Instagram’s impact on well-being among women. It was approved by the University Committee for bioethics research (no. 1/KBBN-UŁ/IV/2018). As we gathered 974 responses, we analyzed them according to search for abnormal values. Due to the fact that the questionnaire was entirely constructed based on the Likert 7 point scale and also free of open questions, we assumed that there is a risk that the respondent will mark all the answers in the same way, which may be an expression of a lack of interest in completing the questionnaire. However, such observations did not appear in the data set. Therefore, we assumed that our collection does not have abnormal data to reject and the final set comprised of 974 records (S2 Dataset).

### Data analysis

For each respondent, levels of self-esteem, life satisfaction, anxiety, depression, and the intensity of Instagram use were calculated. As social comparisons may cause positive or negative effects, they were grouped in the following way: for each record, the values concerning upward identification and downward contrast were added up, as were those for downward identification and upward contrast. A high score indicated whether a respondent was assigned to a “Positive” or “Negative” group. If both scores were the same, the record was labeled “Equal.” The results before and after aggregation are presented in Table 2.

As the empirical research has drawn attention to the issue of common method variance (CMV) and the potential bias it may cause, we decided to include this verification into our data analysis. The CMV’s sources include the use of only one type of item context, respondent, measurement context, and item characteristics, it may also result from certain tendencies while answering a survey on different measures [62–64]. Also a social desirability is a well-known tendency of the respondent that refers to the tendency of respondents to give answers that make them present better [65]. One of the recommended statistical remedies from existing literature is Harman’s Single-Factor Test. It is a post hoc statistical test conducted to investigate the presence of common method effect. In Harman’s single factor score all items are loaded into one common factor. If the total variance for a single factor is less than 50%, it suggests that common method bias doesn’t affect data [66]. We calculated the Harman’s single factor score for our data and obtained a result of 32.78%.

The data were first analyzed in search of possible links in order to justify implementing ANNs. The Shapiro–Wilk test rejected the assumption of normality, so instead of ANOVA, the Kruskal-Wallis test (one-way ANOVA on ranks) was performed. In the next step, a data mining technique–affinity analysis–was used to check co-occurrence relationships. Affinity analysis–association rule mining–is a powerful tool in data mining, used to identifying correlations or patterns [67]. In the final step we implemented and trained a set of ANNs. A classification model of ANN was selected. Table 3 presents all variants included in an implementation process. The data subsets were divided in a following manner: training (70%), testing (15%), and validation (15%). We have performed several different iterations with varying configurations of those settings and then saved the best results.

**Table 3:** Variants of ANNs components used in the implementation.

**Function**	Radial basis function (RBF); Multilayer perceptron (MLP)
**Error function**	Sum of squares; Mutual entropy
**MLP activation function**	Linear; Logistic; Hyperbolic tangent "tanh", Exponential; Sine
**Output neurons function**	Linear; Logistic; Hyperbolic tangent "tanh", Exponential; Sine
**Weights reduction**	In hidden layer; In output layer; None

In the initial phase of network learning, weights and thresholds are given values that are small random numbers. The stopping conditions included the maximum number of epochs, the allowable error rate, the minimum error reduction required in a given learning period and they were set to default values. The number of generated networks in each configuration was set to 1 000, that means that a thousand of random network configuration from all possible ones was automatically built. As for generator settings for initialization, a initial value wasn’t set, so it was provided automatically by Statistica. Network architecture was defined as follows. Number of neurons in an input layer was set to 5 due to the amount of input variables: anxiety, depression, self-esteem, life satisfaction, and Instagram usage intensity. Possible number of neurons in a hidden layer was ranging depending on a network type: MLP from 3 to 500, RBF from 21 to 500 (minimum values set automatically, maximum estimated experimentally in subsequent iterations). Finally, number of neurons in an output layer was varying between 2 or 3 (depending on classified comparison type). All calculations were made using Statistica v.13.3.

This paper presents two powerful, intelligent calculation models, that are two different types of ANNs algorithms, namely multilayer perceptron (MLP) and radial basis function (RBF). MLP consists of basically three layers: an input layer, a hidden layer (one or more), and an output layer [68–70]. Each neuron receives the input data from other neurons and passes through the hidden layers to an output layer. In MLP neurons are interconnected processing nodes to make a network. During training, the MLP starts with small random initial weights values, and then computes a one-pass back propagation algorithm that includes a forward pass propagating the input vector through the network layer by layer, and a backward pass to update the weights by the gradient descent rule [71]. The output of each neuron is a result of weighted set of inputs. Also RBF consists of the same three layers but a hidden layer is single. The input signals are each assigned to a node in the input layer and then passed directly to the hidden one without weights [71]. After processing in hidden layer it goes through output layer, which generates the output data [69,72].

We were using ANN Statistica module in an automatic mode. Thus, the parameters used in training ANN in this mode were limited. We have defined the following sets of values: function describing structure (RBF, MLP), error function (sum of squares and mutual entropy), MLP activation function (linear, logistic, hyperbolic tangent "tanh", exponential, and sine), output neurons function (linear, logistic, hyperbolic tangent "tanh", exponential, and sine), no weights reduction, number of neurons in hidden layer (MLP from 3 to 500, RBF from 21 to 500), number of neurons in an output layer (2 or 3), number of generated networks (1 000). Stopping conditions are set to default values, so without option of modification by the user in an automatic mode. Finally, initial weights were chosen randomly, so we cannot present them explicitly. This is important to highlight, as the results obtained during our analysis may differ when they are re-performed even on the same data set. It results from the use of a random number generator by Statistica, for example when determining the initial weights of neurons. Also, if the decision is made to change the initial value of the random number generator (which we did not modify), it is possible to receive a different division of the available data into a training, test and validation set, which to some extent affects the result of the analysis.

## Results

Descriptive statistics for each type of online social comparison type are presented in Table 4. Respondents labeled as positive (with high levels of upward identification and downward contrast) have the highest scores on Instagram intensity usage. Those marked as negative (downward identification and upward contrast) evinced topmost levels of depression and anxiety and the lowest levels of self-esteem and life satisfaction. Participants that scored equally on positive and negative scale, displayed exactly the opposite tendencies regarding the negative group, and evinced the lowest Instagram intensity usage.

**Table 4:** Descriptive statistics.

Comparison	Positive	Equal	Negative	In all
**N**	566	254	154	974
**Self-esteem (M)**	49.74	51.04	44.19	49.2
**Self-esteem (SD)**	10.11	10.44	8.96	10.26
**Depression (M)**	5.24	4.94	7.49	5.52
**Depression (SD)**	3.41	3.61	3.26	3.54
**Anxiety (M)**	8.54	7.52	9.99	8.5
**Anxiety (SD)**	4.79	5.01	4.08	4.8
**Life satisfaction (M)**	22.38	22.83	21.08	22.29
**Life satisfaction (SD)**	5.87	6.28	5.91	6
**Instagram intensity (M)**	52.96	40.89	50.32	49.4
**Instagram intensity (SD)**	14.11	17.72	14.13	15.97

The Kruskal-Wallis test was performed with the social comparison type (positive, equal, negative) as an independent (grouping) variable with the psychological traits (self-esteem, depression, anxiety, life satisfaction, and the intensity of Instagram use) as the dependent variables (Table 5). The results showed that all *p* values were lower than 0.05, which means that there could be grounds to reject the null hypothesis that all of the population distribution functions are identical. The one-way ANOVA on ranks is an omnibus test statistic and cannot tell which specific groups were statistically significantly different from each other, only that at least two groups were. In our case, this means that the analyzed psychological trait levels for each type of comparison are not equal, so the levels of these traits among different social comparison types differ.

**Table 5:** The Kruskal-Wallis test (one-way ANOVA on ranks) results. Independent (grouping) variable: Social comparison type (positive, equal, negative).

	Kruskal-Wallis test H(2, N = 974)	p value	Positive (N = 566)		Equal (N = 254)		Negative (N = 154)	
			Sum (Rank)	Mean (Rank)	Sum (Rank)	Mean (Rank)	Sum (Rank)	Mean (Rank)
**Self-esteem**	49.33178	.0000	285418.00	504.27	136286.50	536.56	53120.50	344.94
**Depression**	58.85938	.0000	263745.00	465.98	111825.00	440.26	99255.00	644.51
**Anxiety**	27.75889	.0000	277575.00	490.42	108353.50	426.59	88896.50	577.25
**Life satisfaction**	10.48876	.0053	277363.00	490.04	131788.50	518.85	65673.50	426.45
**Instagram intensity**	86.40538	.000	309289.00	546.45	88732.50	349.34	76803.50	498.72

To find more about the associations between social comparison types and the analyzed psychological traits, an affinity analysis was performed as the next step. As this technique requires dichotomous data (1 –if it occurs, 0 –if not present), a database was recalculated so each psychological trait (self-esteem, depression, anxiety, life satisfaction, and the intensity of Instagram use) was given a value of “1” if a score exceeded a sample mean, so was “above average”, and “0” otherwise. The results are described with three coefficients: support, confidence, and lift. “Support” means the probability of the simultaneous occurrence of both traits–“if A then B.” “Confidence” is a conditional probability that an occurrence of one trait (predecessor) will cause an occurrence of another trait (successor). The third measure called the lift or lift ratio is the ratio of confidence to expected confidence. The correlation coefficient is a quotient of the support value and the element from the product of support values, separately for A and B. To present the core association rules, only those with a confidence level above 50% are presented in the results (Table 6).

**Table 6:** Affinity analysis results (with a confidence level above 50%).

Predecessor	==>	Successor	Support (%)	Confidence (%)	Correlation (%)
Self-esteem ⩵ 1	==>	Negative comparison ⩵ 0	45.28	92.45	70.51
Depression ⩵ 0	==>	Negative comparison ⩵ 0	47.33	92.20	72.00
Anxiety ⩵ 0	==>	Negative comparison ⩵ 0	46.20	88.24	69.59
Life satisfaction ⩵ 1	==>	Negative comparison ⩵ 0	45.48	88.07	68.98
Instagram intensity ⩵ 0	==>	Negative comparison ⩵ 0	39.22	85.27	63.03
Instagram intensity ⩵ 1	==>	Equal comparison ⩵ 0	45.07	83.46	71.34
Instagram intensity ⩵ 1	==>	Negative comparison ⩵ 0	44.97	83.27	66.69
Life satisfaction ⩵ 0	==>	Negative comparison ⩵ 0	38.71	80.04	60.66
Anxiety ⩵ 1	==>	Negative comparison ⩵ 0	37.99	79.74	59.98
Anxiety ⩵ 1	==>	Equal comparison ⩵ 0	37.68	79.09	63.50
Self-esteem ⩵ 0	==>	Equal comparison ⩵ 0	39.94	78.27	65.03
Life satisfaction ⩵ 0	==>	Equal comparison ⩵ 0	37.47	77.49	62.68
Depression ⩵ 1	==>	Equal comparison ⩵ 0	37.27	76.58	62.14
Self-esteem ⩵ 0	==>	Negative comparison ⩵ 0	38.91	76.26	59.37
Depression ⩵ 1	==>	Negative comparison ⩵ 0	36.86	75.74	57.58
Depression ⩵ 0	==>	Equal comparison ⩵ 0	36.65	71.40	59.50
Life satisfaction ⩵ 1	==>	Equal comparison ⩵ 0	36.45	70.58	58.99
Self-esteem ⩵ 1	==>	Equal comparison ⩵ 0	33.98	69.39	56.48
Anxiety ⩵ 0	==>	Equal comparison ⩵ 0	36.24	69.22	58.25
Instagram intensity ⩵ 1	==>	Positive comparison ⩵ 1	36.04	66.73	64.33
Depression ⩵ 0	==>	Positive comparison ⩵ 1	32.65	63.60	59.78
Positive Comparison ⩵ 1	==>	Instagram intensity ⩵ 1	36.04	62.01	64.33
Self-esteem ⩵ 1	==>	Positive comparison ⩵ 1	30.29	61.84	56.77
Equal comparison ⩵ 0	==>	Instagram intensity ⩵ 1	45.07	60.97	71.34
Anxiety ⩵ 1	==>	Positive comparison ⩵ 1	28.03	58.84	53.27
Life satisfaction ⩵ 1	==>	Positive comparison ⩵ 1	30.29	58.65	55.29
Life satisfaction ⩵ 0	==>	Positive comparison ⩵ 1	27.82	57.54	52.49
Anxiety ⩵ 0	==>	Positive comparison ⩵ 1	30.08	57.45	54.53
Positive comparison ⩵ 0	==>	Instagram intensity ⩵ 0	23.92	57.11	54.50
Negative comparison ⩵ 0	==>	Depression ⩵ 0	47.33	56.22	72.00
Positive comparison ⩵ 1	==>	Depression ⩵ 0	32.65	56.18	59.78
Positive comparison ⩵ 0	==>	Self-esteem ⩵ 0	23.20	55.39	50.19
Positive comparison ⩵ 0	==>	Depression ⩵ 1	23.20	55.39	51.39
Negative comparison ⩵ 0	==>	Anxiety ⩵ 0	46.20	54.88	69.59
Self-esteem ⩵ 0	==>	Positive comparison ⩵ 1	27.82	54.53	51.10
Equal comparison ⩵ 0	==>	Self-esteem ⩵ 0	39.94	54.03	65.03
Negative comparison ⩵ 0	==>	Life satisfaction ⩵ 1	45.48	54.02	68.98
Negative comparison ⩵ 0	==>	Self-esteem ⩵ 1	45.28	53.78	70.51
Negative comparison ⩵ 0	==>	Instagram intensity ⩵ 1	44.97	53.41	66.69
Positive comparison ⩵ 1	==>	Self-esteem ⩵ 1	30.29	52.12	56.77
Positive comparison ⩵ 1	==>	Life satisfaction ⩵ 1	30.29	52.12	55.29
Instagram intensity ⩵ 0	==>	Positive comparison ⩵ 0	23.92	52.01	54.50
Positive comparison ⩵ 1	==>	Anxiety ⩵ 0	30.08	51.77	54.53
Equal comparison ⩵ 0	==>	Anxiety ⩵ 1	37.68	50.97	63.50
Equal comparison ⩵ 0	==>	Life satisfaction ⩵ 0	37.47	50.69	62.68
Equal comparison ⩵ 0	==>	Depression ⩵ 1	37.27	50.42	62.14

The above rules presented essentially that respondents possessing high self-esteem were more likely to experience positive comparisons than negative ones. High levels of depression or anxiety diminished equal comparison types, though people scoring strong on the latter had also the tendency to positive comparisons. Being satisfied with one’s life implied positive and reduced possibility of negative comparisons. Vast Instagram usage was associated with positive comparisons as well. Also positive comparison, as a predecessor variable, implicated low depression and anxiety as well as high self-esteem, life satisfaction and Instagram usage intensity.

The one-way ANOVA on ranks and association rule mining gave grounds to state that there are relationships between the analyzed psychological features and social comparison types, which justifies the construction of ANNs on the basis of the collected data. The first ANN model had an output neuron with one type of social comparison variable. In this case, the ANN was able to predict whether a respondent was going to indicate a positive, negative, or equal style of social comparison on Instagram. Unfortunately, this model achieved only average quality testing results, amounting to 61.64%. Quality of testing is calculated for classification networks as the relative number of cases correctly classified (relative to the total number of cases), so this value means that less than two-thirds of all cases were classified correctly. We concluded that these results were unsatisfactory, and another approach was implemented. The second model comprised three independent ANNs, each predicting the possibility of a positive, negative, or equal social comparison. In this model, the results were more satisfactory, with quality of testing varying from 71% to 82%. One ANN model, in particular, proved to be the most fitting: an RBF 5-100-2 network, with an RBFT learning algorithm, cross-entropy error function, Gaussian activation function in a hidden layer, and Softmax activation function in an output layer. The values 5, 100, and 2 are the numbers of neurons in the input, hidden, and output layers, respectively. The best network predicting three types of comparisons was RBF 5-25-3, that means it possessed 25 neurons in a hidden layer, while new networks structures were RBF/MLP 5-100-2. This amount was automatically estimated by a software. A huge number of hidden neurons may cause network overfitting that means some nodes are unnecessary [73]. Overfitting occurs when the performance on test set is much lower than the performance on learning set due to the fact that the model fits too much to seen data, and do not generalize well. As our results provided higher testing results comparing to learning outcomes, overfitting wasn’t recorded in our networks. This is coherent with the approach that nowadays the trend is to design artificial neural networks with more hidden neurons, and it depends solely on data particularities [74]. RBFT is the default learning algorithm for RBF networks in Statistica. The cross-entropy error function assumes that data comes from a family of exponential distributions and provides a direct probabilistic interpretation of network outputs. Cross entropy is used only for classification networks. If this type of error function is selected, the activation function in an output layer will always be set to Softmax; it is a specialized activation function adapted to classification problems in which the representation of a one-out-of-N output variable is used. Softmax sets the normalized values of the exponential function (add up to unity). In conjunction with the error function based on mutual entropy, it is possible to estimate the likelihood of belonging to particular classes by means of a perceptron [75]. Finally, for an RBF network, the activation function for the hidden layer is automatically set to Gauss.

For a model predicting equal social comparison, a MLP model was also calculated as the most powerful. The network was formed as an MLP 5-100-2, with a Broyden-Fletcher-Goldfarb-Shanno (BFGS) 8 learning algorithm. It means that this algorithm was used to optimize the weights of the network and that the learning process required 8 epochs (learning cycles). BFGS is of the most recommended techniques used by STATISTICA for training neural networks. It performs significantly better than the more traditional algorithms such as Gradient Descent but it is more memory intensive and computationally demanding. Nonetheless, BFGS may require a smaller number of iterations to train a neural network given their fast convergence rate and more intelligent search criterion. This model used logistic activation function in the hidden layer and Tanh in the output layer. Sum-of-squares was selected as the error function during network training process. Still, the second model for the equal type of social comparison, with only slightly worse quality of testing (80.14 compared to 80.82 for MLP), was the aforementioned RBF network.

To summarize this part on ANNs performance, let’s start with the fact that MLP and RBF belong to a general class of neural networks called feed-forward, where the information processing follows one direction from the input to output neurons. Despite their similarities in structure, the are several vast differences. RBFs with a single hidden layer (the structure used by Statistica) are usually simpler and easier to train than MLPs due to their three-layer structure. In MLPs outputs are decided by all the neurons. Since RBF networks use only fixed basis functions, their representation power could be largely restricted. When multilayer RBF networks are used more efficient local approximation is possible and other advantages in generalization and training, unfortunately this option isn’t offered by Statistica so far [76]. Both MLP and RBF possess also different classification techniques, which are hyper surfaces and hyper spheres accordingly [70,77]. RBFs are widely used in various fields such as function approximation and pattern classification (the latter one is important for our ANNs design). Among the most important advantages of RBF following should be mentioned: easy design, very strong tolerance to input noise, fast and comprehensive training, responding well to new patterns absent in learning [77–81]. Though, RBFs are usually slower and larger than MLPs, and they often have weaker performance. RBFs also have lower performance than MLPs, especially with a large number of input variables (they are more sensitive to switching on unnecessary inputs). In our study, RBF networks operated better in most cases, the only one where MLP scored better was a network with equal comparison output. As RBF perform well in classification tasks, such results seem to be reasonable (Table 7).

**Table 7:** Summary of ANNs.

**ANNs with comparison type output: positive, negative, equal**								
ID	Name	Classification accuracy (learning) (%)	Classification accuracy (testing) (%)	Classification accuracy (validation) (%)	Learning algorithm	Error function	Activation function (hidden layer)	Activation function (output layer)
88	RBF 5-25-3	63.78	61.64	59.59	RBFT	Cross entropy	Gaussian	Softmax
315	RBF 5-26-3	64.66	61.64	57.53	RBFT	Cross entropy	Gaussian	Softmax
103	RBF 5-29-3	65.69	60.96	60.96	RBFT	Cross entropy	Gaussian	Softmax
192	RBF 5-23-3	61.14	60.96	59.59	RBFT	Cross entropy	Gaussian	Softmax
329	RBF 5-21-3	57.77	60.96	52.74	RBFT	Cross entropy	Gaussian	Softmax
**ANNs with positive comparison output**								
ID	Name	Classification accuracy (learning) (%)	Classification accuracy (testing) (%)	Classification accuracy (validation) (%)	Learning algorithm	Error function	Activation function (hidden layer)	Activation function (output layer)
669	RBF 5-100-2	53.81	71.23	55.48	RBFT	Cross entropy	Gaussian	Softmax
981	RBF 5-100-2	61.44	71.23	54.11	RBFT	Cross entropy	Gaussian	Softmax
944	RBF 5-100-2	62.61	70.55	54.79	RBFT	Cross entropy	Gaussian	Softmax
593	RBF 5-100-2	56.74	69.18	52.74	RBFT	Cross entropy	Gaussian	Softmax
822	RBF 5-100-2	61.88	69.18	56.85	RBFT	Cross entropy	Gaussian	Softmax
**ANNs with negative comparison output**								
ID	Name	Classification accuracy (learning) (%)	Classification accuracy (testing) (%)	Classification accuracy (validation) (%)	Learning algorithm	Error function	Activation function (hidden layer)	Activation function (output layer)
777	RBF 5-100-2	80.94	82.88	79.45	RBFT	Cross entropy	Gaussian	Softmax
589	RBF 5-100-2	84.46	82.19	81.51	RBFT	Cross entropy	Gaussian	Softmax
624	RBF 5-100-2	87.68	82.19	82.19	RBFT	Cross entropy	Gaussian	Softmax
634	RBF 5-100-2	85.63	82.19	82.88	RBFT	Sum-of-squares	Gaussian	Linear
678	RBF 5-100-2	86.07	82.19	84.25	RBFT	Sum-of-squares	Gaussian	Linear
**ANNs with equal comparison output**								
ID	Name	Classification accuracy (learning)	Classification accuracy (testing)	Classification accuracy (validation)	Learning algorithm	Error function	Activation function (hidden layer)	Activation function (output layer)
167	MLP 5-100-2	76.98	80.82	74.66	BFGS 8	Sum-of-squares	Logistic	Tanh
24	RBF 5-100-2	80.06	80.14	75.34	RBFT	Sum-of-squares	Gaussian	Linear
69	MLP 5-100-2	77.27	80.14	74.66	BFGS 8	Sum-of-squares	Logistic	Sinus
206	MLP 5-100-2	77.27	80.14	74.66	BFGS 8	Sum-of-squares	Logistic	Tanh
437	MLP 5-100-2	76.83	80.14	74.66	BFGS 7	Cross entropy	Logistic	Softmax

## Discussion

Social media may cause negative effects on women’s mood and body image, raising concerns about appearance and lowering self-esteem, well-being, as well as life satisfaction [82]. This paper was aiming at studying whether psychological characteristics, including self-esteem, life satisfaction, depression, anxiety, and intensity of Instagram use could be related to social comparisons made by young women on Instagram. The one-way ANOVA on ranks results showed that there is a significant difference between the studied groups (positive, negative, and equal social comparison types). However, it does not provide any information about which groups are different. To find more about the possible directions of associations between the social comparison types and the analyzed psychological traits, an affinity analysis was performed. Among the links with the highest confidence level, the following can be mentioned. The occurrence of high self-esteem and life satisfaction, as well as low levels of anxiety and depression, all resulted in there not being a negative social comparison. The equal type of social comparison was described by dissociation rules concerning high anxiety and depression, and low self-esteem and life satisfaction; that means that these traits also did not produce this comparison type. The positive comparison type was associated with high Instagram intensity usage, low depression, and high life satisfaction and self-esteem. Concerning the direction of association between the psychological traits and social comparison types, these results suggest that having different levels of these psychological traits may cause different social comparison types rather than vice versa.

The second aim was to test the possibility of implementing ANNs in psychological data to predict a type of social comparison. The results show that three models using binary classification have quality of the predictions as it ranged between 71% to 82% of all analyzed cases. This result may be considered acceptable, taking into consideration that an ANN operation will only be as good as the strength of the associations between the variables. The type of ANN that turned out best (RBF) was quite surprising, however. MLP is the most common type of network; it can be time-consuming but the received networks are small, fast, and they give better results than other types of networks. RBF networks are usually slower and larger than MLPs, and they often have weaker performance, especially with a large number of input variables. The justification of the better results of the RBF networks may be that the input layer consisted of only five neurons, so they would not weaken their performance significantly.

The study has several limitations. First, all the data were gathered by questionnaires completed by the respondents. It should be emphasized that self-assessment scales are only valid for screening purposes; a definitive diagnosis must rest on a clinical examination. Second, associations between the variables existed, but–in several cases–the affinity analysis proved that the opposite association rules scored similar confidence levels. One such example was the influence of life satisfaction on the positive social comparison type. A high level of life satisfaction indicated a positive social comparison, with a confidence level at 58.65%; a low level of this trait was indicated at 57.54%. Finally, the strength of associations influenced the operation of the ANNs, scoring a maximum of 82% for its effectiveness.

Despite the limitations, the study showed that psychological traits like self-esteem, life satisfaction, depression, anxiety, and the intensity of Instagram use may influence the way young women from the studied sample compared themselves while using this social medium. The results also supported the implementation of ANNs in psychological studies. As human behavioral analysis has been a subject of study in various fields of science, including sociology, psychology, and computer science, an increased interest in artificial intelligence methods for analyzing behavioral data in psychology can also be noticed [14,15]. Among the avenues of future research, the set of analyzed psychological traits could be broadened, and male respondents could be included as a control group to investigate whether these associations are typical for women or if they are independent of gender.

## Supporting information

S1 Table CiteSpace clusters on social comparisons online research from 2014–2019 (Web of Science). (DOCX)

S1 Appendix Manuscripts in clusters (created automatically by CiteSpace). (DOCX)

S2 Appendix CiteSpace analysis criteria. (DOCX)

S3 Appendix Questionnaire (in English and Polish). (DOCX)

S1 Dataset CiteSpace input text file. (ZIP)

S2 Dataset Raw data from the questionnaire. (XLSX)
