# Supplementary material for: Artificial neural networks for predicting social comparison effects among female Instagram users
Source: PLoS One. 2020 Feb 25;15(2):e0229354. doi: 10.1371/journal.pone.0229354 (PMC7041802; doi:10.1371/journal.pone.0229354)
Supplement: S1 Table — (DOCX) [file pone.0229354.s001.docx]

# Table 1. CiteSpace clusters on social comparisons online research from 2014-2019 (Web of Science).

| **ID** | **Silhouette** | **Size** | **Mean (Year)** | **Label** |
| --- | --- | --- | --- | --- |
| 0 | 0.85 | 32 | 2016 | effect; instagram; body dissatisfaction; social comparison; women; sociocultural model; predictors; young women; instagrammers' body image; appearance ideal \| thinspiration; comparison; twitter; fitspiration communities; tweeting weight loss; sociocultural model; predictors; young women; instagrammers' body image; appearance ideal |
| 1 | 0.761 | 31 | 2015 | social comparison; subjective well-being; facebook; impact; social media use; personality traits; communication type; exploring causes; envy; don't \| instagram use; motives; cross-cultural comparison; exploring cultural differences; american social network sites; envy; facebook depression'; moderating roles; orthorexia nervosa; symptoms |
| 2 | 0.841 | 30 | 2016 | social media; millennial population; examination; social interaction; specific social media behaviors; major depressive disorder; associations; college; envy; identity distress \| social comparison; facebook; interplay; psychological well-being; faces; associations; college; envy; identity distress; university |
| 3 | 0.915 | 24 | 2014 | social media; social comparison; body image; pinterest; thinterest; facebook use; self-comparison; body image dissatisfaction; pregnancy; role \| facebook use; self-comparison; body image dissatisfaction; pregnancy; role; mood; facebook; social comparisons; impact; potential moderators |
| 5 | 0.886 | 9 | 2014 | internet support group; direction; randomized trial; online expressive writing; people; anxiety; depression; young women; predictors; anonymous online settings \| social network sites'; ssci journals; review; educational use; publications; research trends; young women; predictors; anonymous online settings; chambers |

Note. These are results produced by CiteSpace automatically. The ID column represents the cluster number automatically assigned by CiteSpace. The average year of publication indicates whether it is formed by generally recent or old papers, while the silhouette column shows the homogeneity of a cluster. The higher the silhouette score (its value ranges between -1 and 1), the more consistent the cluster members are. Labels are built to characterize the nature of an identified cluster by extracting noun phrases from the titles, keyword lists, or abstracts of articles that cited the particular cluster.
