# Supplementary material for: Artificial neural networks for predicting social comparison effects among female Instagram users
Source: PLoS One. 2020 Feb 25;15(2):e0229354. doi: 10.1371/journal.pone.0229354 (PMC7041802; doi:10.1371/journal.pone.0229354)
Supplement: S1 Appendix — (DOCX) [file pone.0229354.s002.docx]

# Manuscripts in clusters (created automatically by CiteSpace)

Cluster 1

| Tamplin, Natalie C (2018) Social media literacy protects against the negative impact of exposure to appearance ideal social media images in young adult women but not men. BODY IMAGE DOI 10.1016/j.bodyim.2018.05.003 |
| --- |
| Alberga, Angela S (2018) Fitspiration and thinspiration: a comparison across three social networking sites. JOURNAL OF EATING DISORDERS DOI 10.1186/s40337-018-0227-x |
| Powell, Elisabeth (2018) Attachment security and social comparisons as predictors of pinterest users' body image concerns. COMPUTERS IN HUMAN BEHAVIOR DOI 10.1016/j.chb.2018.01.039 |
| Fardouly, Jasmine (2018) Social media is not real life: the effect of attaching disclaimer-type labels to idealized social media images on women's body image and mood. NEW MEDIA & SOCIETY, V20, P18 DOI 10.1177/1461444818771083 |
| Tiggemann, Marika (2018) Tweeting weight loss: a comparison of #thinspiration and #fitspiration communities on twitter. BODY IMAGE DOI 10.1016/j.bodyim.2018.03.002 |
| Saunders, Jessica F (2018) Snaps, selfies, and shares: how three popular social media platforms contribute to the sociocultural model of disordered eating among young women. CYBERPSYCHOLOGY BEHAVIOR AND SOCIAL NETWORKING, V21, P12 DOI 10.1089/cyber.2017.0713 |
| Jin, Seunga Venus (2018) Dieting 2.0!: moderating effects of instagrammers' body image and instafame on other instagrammers' dieting intention. COMPUTERS IN HUMAN BEHAVIOR, V87, P14 DOI 10.1016/j.chb.2018.06.001 |
| Utz, Sonja (2018) Your co-author received 150 citations: pride, but not envy, mediates the effect of system-generated achievement messages on motivation. FRONTIERS IN PSYCHOLOGY DOI 10.3389/fpsyg.2018.00628 |
| Fardouly, Jasmine (2018) Instagram use and young women's body image concerns and self-objectification: testing mediational pathways. NEW MEDIA & SOCIETY, V20, P16 DOI 10.1177/1461444817694499 |
| Tiggemann, Marika (2018) The effect of instagram "likes" on women's social comparison and body dissatisfaction. BODY IMAGE DOI 10.1016/j.bodyim.2018.07.002 |

Cluster 2

| Weinstein, Emily (2017) Adolescents' differential responses to social media browsing: exploring causes and consequences for intervention. COMPUTERS IN HUMAN BEHAVIOR, V76, P10 DOI 10.1016/j.chb.2017.07.038 |
| --- |
| Verduyn, Philippe (2017) Do social network sites enhance or undermine subjective well-being? a critical review. SOCIAL ISSUES AND POLICY REVIEW, V11, P29 DOI 10.1111/sipr.12033 |
| Underwood, Marion K (2017) The power and the pain of adolescents' digital communication: cyber victimization and the perils of lurking. AMERICAN PSYCHOLOGIST, V72, P15 DOI 10.1037/a0040429 |
| Turner, Pixie G (2017) Instagram use is linked to increased symptoms of orthorexia nervosa. EATING AND WEIGHT DISORDERS-STUDIES ON ANOREXIA BULIMIA AND OBESITY DOI 10.1007/s40519-017-0364-2 |
| Yang, Chia-chen (2016) Instagram use, loneliness, and social comparison orientation: interact and browse on social media, but don't compare. CYBERPSYCHOLOGY BEHAVIOR AND SOCIAL NETWORKING DOI 10.1089/cyber.2016.0201 |
| Burke, Moira (2016) The relationship between facebook use and well-being depends on communication type and tie strength. JOURNAL OF COMPUTER-MEDIATED COMMUNICATION, V21, P17 DOI 10.1111/jcc4.12162 |
| Gerson, Jennifer (2016) Subjective well-being and social media use: do personality traits moderate the impact of social comparison on facebook?. COMPUTERS IN HUMAN BEHAVIOR, V63, P10 DOI 10.1016/j.chb.2016.06.023 |
| Chow, Tak Sang (2017) Is there any 'facebook depression'? exploring the moderating roles of neuroticism, facebook social comparison and envy. PERSONALITY AND INDIVIDUAL DIFFERENCES DOI 10.1016/j.paid.2017.07.032 |
| Sheldon, Pavica (2017) A cross-cultural comparison of croatian and american social network sites: exploring cultural differences in motives for instagram use. COMPUTERS IN HUMAN BEHAVIOR DOI 10.1016/j.chb.2017.06.009 |
| Ouwerkerk, Jaap W (2016) Motives for online friending and following: the dark side of social network site connections. SOCIAL MEDIA + SOCIETY DOI 10.1177/2056305116664219 |

Cluster 3

| Chae, Jiyoung (2018) Reexamining the relationship between social media and happiness: the effects of various social media platforms on reconceptualized happiness. TELEMATICS AND INFORMATICS DOI 10.1016/j.tele.2018.04.011 |
| --- |
| Meier, Adrian (2018) Positive side of social comparison on social network sites: how envy can drive inspiration on instagram. CYBERPSYCHOLOGY BEHAVIOR AND SOCIAL NETWORKING DOI 10.1089/cyber.2017.0708 |
| Yang, Chia-chen (2018) Social media social comparison of ability (but not opinion) predicts lower identity clarity: identity processing style as a mediator. JOURNAL OF YOUTH AND ADOLESCENCE, V47, P15 DOI 10.1007/s10964-017-0801-6 |
| Robinson, Anthony (2019) Social comparisons, social media addiction, and social interaction: an examination of specific social media behaviors related to major depressive disorder in a millennial population. JOURNAL OF APPLIED BIOBEHAVIORAL RESEARCH DOI 10.1111/jabr.12158 |
| Yang, Chia-Chen (2018) Not necessarily detrimental: two social comparison orientations and their associations with social media use and college social adjustment. COMPUTERS IN HUMAN BEHAVIOR DOI 10.1016/j.chb.2018.02.020 |
| Yang, Chia-chen (2018) Social media social comparison and identity distress at the college transition: a dual-path model. JOURNAL OF ADOLESCENCE, V69, P11 DOI 10.1016/j.adolescence.2018.09.007 |
| Park, Sun Young (2018) Two faces of social comparison on facebook: the interplay between social comparison orientation, emotions, and psychological well-being. COMPUTERS IN HUMAN BEHAVIOR, V79, P11 DOI 10.1016/j.chb.2017.10.028 |
| Mingoia, John (2017) The relationship between social networking site use and the internalization of a thin ideal in females: a meta-analytic review. FRONTIERS IN PSYCHOLOGY DOI 10.3389/fpsyg.2017.01351 |
| Thomas, Lisa (2017) Understanding social media and identity work in young people transitioning to university. COMPUTERS IN HUMAN BEHAVIOR, V76, P13 DOI 10.1016/j.chb.2017.08.021 |

Cluster 4

| Fardouly, Jasmine (2015) Social comparisons on social media: the impact of facebook on young women's body image concerns and mood. BODY IMAGE DOI 10.1016/j.bodyim.2014.12.002 |
| --- |
| Fardouly, Jasmine (2016) Social media and body image concerns: current research and future directions. CURRENT OPINION IN PSYCHOLOGY DOI 10.1016/j.copsyc.2015.09.005 |
| Tiggemann, Marika (2015) "exercise to be fit, not skinny": the effect of fitspiration imagery on women's body image. BODY IMAGE DOI 10.1016/j.bodyim.2015.06.003 |
| Lup, Katerina (2015) Instagram #instasad?: exploring associations among instagram use, depressive symptoms, negative social comparison, and strangers followed. CYBERPSYCHOLOGY BEHAVIOR AND SOCIAL NETWORKING DOI 10.1089/cyber.2014.0560 |
| Walker, Morgan (2015) Facebook use and disordered eating in college-aged women. JOURNAL OF ADOLESCENT HEALTH DOI 10.1016/j.jadohealth.2015.04.026 |
| Hicks, S (2016) Higher facebook use predicts greater body image dissatisfaction during pregnancy: the role of self-comparison. MIDWIFERY DOI 10.1016/j.midw.2016.06.018 |
| Kim, Ji Won (2015) Body image 2.0: associations between social grooming on facebook and body image concerns. COMPUTERS IN HUMAN BEHAVIOR DOI 10.1016/j.chb.2015.01.009 |
| Fardouly, Jasmine (2015) The mediating role of appearance comparisons in the relationship between media usage and self-objectification in young women. PSYCHOLOGY OF WOMEN QUARTERLY, V39, P11 DOI 10.1177/0361684315581841 |
| Arroyo, Analisa (2016) Negative body talk as an outcome of friends' fitness posts on social networking sites: body surveillance and social comparison as potential moderators. JOURNAL OF APPLIED COMMUNICATION RESEARCH, V44, P20 DOI 10.1080/00909882.2016.1192293 |
| Lewallen, Jennifer (2016) Pinterest or thinterest?: social comparison and body image on social media. SOCIAL MEDIA + SOCIETY DOI 10.1177/2056305116640559 |

Cluster 5

| Akcayir, Gokce (2016) Research trends in social network sites' educational use: a review of publications in all ssci journals to 2015. REVIEW OF EDUCATION DOI 10.1002/rev3.3075 |
| --- |
| Grion, Valentina (2016) Social network sites in secondary school: when students and teachers express their viewpoints. ICERI2016: 9TH INTERNATIONAL CONFERENCE OF EDUCATION, RESEARCH AND INNOVATION |
| Burnell, Kaitlyn (2016) Predictors of mobile phone and social networking site dependency in adulthood. CYBERPSYCHOLOGY BEHAVIOR AND SOCIAL NETWORKING DOI 10.1089/cyber.2016.0209 |
| Balakrishnan, Vimala (2016) Students' learning styles and their effects on the use of social media technology for learning. TELEMATICS AND INFORMATICS, V33, P14 DOI 10.1016/j.tele.2015.12.004 |
| Hayat, Tsahi (2017) "you too, second screeners?" second screeners' echo chambers during the 2016 us elections primaries. JOURNAL OF BROADCASTING & ELECTRONIC MEDIA, V61, P18 DOI 10.1080/08838151.2017.1309417 |
| Martincic-Ipsic, Sanda (2017) Link prediction on twitter. PLOS ONE DOI 10.1371/journal.pone.0181079 |
| Andrew, Rachel (2016) Predicting body appreciation in young women: an integrated model of positive body image. BODY IMAGE DOI 10.1016/j.bodyim.2016.04.003 |
| Dean, Jeremy (2016) Direction to an internet support group compared with online expressive writing for people with depression and anxiety: a randomized trial. JMIR MENTAL HEALTH DOI 10.2196/mental.5133 |
| Doering, Nicola (2016) How gender-stereotypical are selfies? a content analysis and comparison with magazine adverts. COMPUTERS IN HUMAN BEHAVIOR DOI 10.1016/j.chb.2015.10.001 |
| Barnidge, Matthew (2017) Exposure to political disagreement in social media versus face-to-face and anonymous online settings. POLITICAL COMMUNICATION, V34, P20 DOI 10.1080/10584609.2016.1235639 |
