# Supplementary material for: Artificial neural networks for predicting social comparison effects among female Instagram users
Source: PLoS One. 2020 Feb 25;15(2):e0229354. doi: 10.1371/journal.pone.0229354 (PMC7041802; doi:10.1371/journal.pone.0229354)
Supplement: S2 Appendix — (DOCX) [file pone.0229354.s003.docx]

# CiteSpace analysis criteria

**Search terms**: online social comparisons, social comparisons on Instagram, social comparisons on social media

**Inclusion criteria**: publication date: 2014 and 2019; indexed in Web of Science

**Exclusion criteria**: publications before 2014

**Search result:** S2. Appendix. Manuscripts in clusters (created automatically by CiteSpace)
