# Supplementary material for: Artificial neural networks for predicting social comparison effects among female Instagram users
Source: PLoS One. 2020 Feb 25;15(2):e0229354. doi: 10.1371/journal.pone.0229354 (PMC7041802; doi:10.1371/journal.pone.0229354)
Supplement: S3 Appendix — (DOCX) [file pone.0229354.s004.docx]

# Questionnaire (English version)

Part I. Instagram usage.

1. If I could visit only one site on the Internet, it would be Instagram.
   1. I totally agree
   2. I agree very much
   3. I rather agree
   4. No opinion
   5. I rather disagree
   6. I disagree very much
   7. I totally disagree
2. I feel bad if I don't check my Instagram daily.
   1. I totally agree
   2. I agree very much
   3. I rather agree
   4. No opinion
   5. I rather disagree
   6. I disagree very much
   7. I totally disagree
3. I often search for Internet connection in order to visit Instagram.
   1. I totally agree
   2. I agree very much
   3. I rather agree
   4. No opinion
   5. I rather disagree
   6. I disagree very much
   7. I totally disagree
4. Before going to sleep, I check Instagram once more.
   1. I totally agree
   2. I agree very much
   3. I rather agree
   4. No opinion
   5. I rather disagree
   6. I disagree very much
   7. I totally disagree
5. Watching Instagram posts is good for overcoming boredom.
   1. I totally agree
   2. I agree very much
   3. I rather agree
   4. No opinion
   5. I rather disagree
   6. I disagree very much
   7. I totally disagree
6. When I'm bored, I often go to Instagram.
   1. I totally agree
   2. I agree very much
   3. I rather agree
   4. No opinion
   5. I rather disagree
   6. I disagree very much
   7. I totally disagree
7. If I'm bored, I open Instagram.
   1. I totally agree
   2. I agree very much
   3. I rather agree
   4. No opinion
   5. I rather disagree
   6. I disagree very much
   7. I totally disagree
8. I spent time on Instagram at the expense of my obligations.
   1. I totally agree
   2. I agree very much
   3. I rather agree
   4. No opinion
   5. I rather disagree
   6. I disagree very much
   7. I totally disagree
9. I spend more time on Instagram than I would like to.
   1. I totally agree
   2. I agree very much
   3. I rather agree
   4. No opinion
   5. I rather disagree
   6. I disagree very much
   7. I totally disagree
10. It happens that I use Instagram instead of sleeping.
    1. I totally agree
    2. I agree very much
    3. I rather agree
    4. No opinion
    5. I rather disagree
    6. I disagree very much
    7. I totally disagree
11. My Instagram profile is rather detailed.
    1. I totally agree
    2. I agree very much
    3. I rather agree
    4. No opinion
    5. I rather disagree
    6. I disagree very much
    7. I totally disagree
12. I like refining my Instagram profile.
    1. I totally agree
    2. I agree very much
    3. I rather agree
    4. No opinion
    5. I rather disagree
    6. I disagree very much
    7. I totally disagree
13. It is important for me to update my Instagram profile regularly.
    1. I totally agree
    2. I agree very much
    3. I rather agree
    4. No opinion
    5. I rather disagree
    6. I disagree very much
    7. I totally disagree
14. When I compare my Instagram profile to people who have better profiles, I feel that it is possible that one day I will also reach their level of popularity.
    1. I totally agree
    2. I agree very much
    3. I rather agree
    4. No opinion
    5. I rather disagree
    6. I disagree very much
    7. I totally disagree
15. When I compare my Instagram profile to people who have better profiles, I hope my profile will improve.
    1. I totally agree
    2. I agree very much
    3. I rather agree
    4. No opinion
    5. I rather disagree
    6. I disagree very much
    7. I totally disagree
16. When I compare my Instagram profile to people who have better profiles, I'm happy that my profile can also be so good.
    1. I totally agree
    2. I agree very much
    3. I rather agree
    4. No opinion
    5. I rather disagree
    6. I disagree very much
    7. I totally disagree
17. When I compare my Instagram profile to people who have better profiles, I feel frustrated with the level of my own profile.
    1. I totally agree
    2. I agree very much
    3. I rather agree
    4. No opinion
    5. I rather disagree
    6. I disagree very much
    7. I totally disagree
18. When I compare my Instagram profile to people who have better profiles, I feel anxious that my profile is not achieving such results.
    1. I totally agree
    2. I agree very much
    3. I rather agree
    4. No opinion
    5. I rather disagree
    6. I disagree very much
    7. I totally disagree
19. When I compare my Instagram profile to people who have better profiles, I get depressed realizing that my profile is not so good.
    1. I totally agree
    2. I agree very much
    3. I rather agree
    4. No opinion
    5. I rather disagree
    6. I disagree very much
    7. I totally disagree
20. When I compare my Instagram profile to people who have weaker profiles, I'm afraid my profile will get worse.
    1. I totally agree
    2. I agree very much
    3. I rather agree
    4. No opinion
    5. I rather disagree
    6. I disagree very much
    7. I totally disagree
21. When I compare my Instagram profile to people who have weaker profiles, I'm afraid that the future of my profile will be the same.
    1. I totally agree
    2. I agree very much
    3. I rather agree
    4. No opinion
    5. I rather disagree
    6. I disagree very much
    7. I totally disagree
22. When I compare my Instagram profile to people who have weaker profiles, I'm afraid my profile's popularity will drop.
    1. I totally agree
    2. I agree very much
    3. I rather agree
    4. No opinion
    5. I rather disagree
    6. I disagree very much
    7. I totally disagree
23. When I compare my Instagram profile to people who have weaker profiles, I feel how good I am doing.
    1. I totally agree
    2. I agree very much
    3. I rather agree
    4. No opinion
    5. I rather disagree
    6. I disagree very much
    7. I totally disagree
24. When I compare my Instagram profile to people who have weaker profiles, I feel relief about my own profile.
    1. I totally agree
    2. I agree very much
    3. I rather agree
    4. No opinion
    5. I rather disagree
    6. I disagree very much
    7. I totally disagree
25. When I compare my Instagram profile to people who have weaker profiles, I'm happy I'm doing so well.
    1. I totally agree
    2. I agree very much
    3. I rather agree
    4. No opinion
    5. I rather disagree
    6. I disagree very much
    7. I totally disagree
26. Part II. Well-being.
27. On the whole, I am satisfied with myself.
    1. I totally agree
    2. I agree very much
    3. I rather agree
    4. No opinion
    5. I rather disagree
    6. I disagree very much
    7. I totally disagree
28. At times I think I am no good at all.
    1. I totally agree
    2. I agree very much
    3. I rather agree
    4. No opinion
    5. I rather disagree
    6. I disagree very much
    7. I totally disagree
29. I feel that I have a number of good qualities.
    1. I totally agree
    2. I agree very much
    3. I rather agree
    4. No opinion
    5. I rather disagree
    6. I disagree very much
    7. I totally disagree
30. I am able to do things as well as most other people.
    1. I totally agree
    2. I agree very much
    3. I rather agree
    4. No opinion
    5. I rather disagree
    6. I disagree very much
    7. I totally disagree
31. I feel I do not have much to be proud of.
    1. I totally agree
    2. I agree very much
    3. I rather agree
    4. No opinion
    5. I rather disagree
    6. I disagree very much
    7. I totally disagree
32. I certainly feel useless at times.
    1. I totally agree
    2. I agree very much
    3. I rather agree
    4. No opinion
    5. I rather disagree
    6. I disagree very much
    7. I totally disagree
33. I feel that I'm a person of worth, at least on an equal plane with others.
    1. I totally agree
    2. I agree very much
    3. I rather agree
    4. No opinion
    5. I rather disagree
    6. I disagree very much
    7. I totally disagree
34. I wish I could have more respect for myself.
    1. I totally agree
    2. I agree very much
    3. I rather agree
    4. No opinion
    5. I rather disagree
    6. I disagree very much
    7. I totally disagree
35. All in all, I am inclined to feel that I am a failure.
    1. I totally agree
    2. I agree very much
    3. I rather agree
    4. No opinion
    5. I rather disagree
    6. I disagree very much
    7. I totally disagree
36. I take a positive attitude toward myself.
    1. I totally agree
    2. I agree very much
    3. I rather agree
    4. No opinion
    5. I rather disagree
    6. I disagree very much
    7. I totally disagree
37. I feel tense or 'wound up'.
    1. I totally agree
    2. I agree very much
    3. I rather agree
    4. No opinion
    5. I rather disagree
    6. I disagree very much
    7. I totally disagree
38. I still enjoy the things I used to enjoy.
    1. I totally agree
    2. I agree very much
    3. I rather agree
    4. No opinion
    5. I rather disagree
    6. I disagree very much
    7. I totally disagree
39. I get a sort of frightened feeling as if something awful is about to happen.
    1. I totally agree
    2. I agree very much
    3. I rather agree
    4. No opinion
    5. I rather disagree
    6. I disagree very much
    7. I totally disagree
40. I can laugh and see the funny side of things.
    1. I totally agree
    2. I agree very much
    3. I rather agree
    4. No opinion
    5. I rather disagree
    6. I disagree very much
    7. I totally disagree
41. Worrying thoughts go through my mind.
    1. I totally agree
    2. I agree very much
    3. I rather agree
    4. No opinion
    5. I rather disagree
    6. I disagree very much
    7. I totally disagree
42. I feel cheerful.
    1. I totally agree
    2. I agree very much
    3. I rather agree
    4. No opinion
    5. I rather disagree
    6. I disagree very much
    7. I totally disagree
43. I can sit at ease and feel relaxed.
    1. I totally agree
    2. I agree very much
    3. I rather agree
    4. No opinion
    5. I rather disagree
    6. I disagree very much
    7. I totally disagree
44. I feel as if I am slowed down.
    1. I totally agree
    2. I agree very much
    3. I rather agree
    4. No opinion
    5. I rather disagree
    6. I disagree very much
    7. I totally disagree
45. I get a sort of frightened feeling like 'butterflies' in the stomach.
    1. I totally agree
    2. I agree very much
    3. I rather agree
    4. No opinion
    5. I rather disagree
    6. I disagree very much
    7. I totally disagree
46. I have lost interest in my appearance.
    1. I totally agree
    2. I agree very much
    3. I rather agree
    4. No opinion
    5. I rather disagree
    6. I disagree very much
    7. I totally disagree
47. I feel restless as I have to be on the move.
    1. I totally agree
    2. I agree very much
    3. I rather agree
    4. No opinion
    5. I rather disagree
    6. I disagree very much
    7. I totally disagree
48. I look forward with enjoyment to things.
    1. I totally agree
    2. I agree very much
    3. I rather agree
    4. No opinion
    5. I rather disagree
    6. I disagree very much
    7. I totally disagree
49. I get sudden feelings of panic.
    1. I totally agree
    2. I agree very much
    3. I rather agree
    4. No opinion
    5. I rather disagree
    6. I disagree very much
    7. I totally disagree
50. I can enjoy a good book or radio or TV program.
    1. I totally agree
    2. I agree very much
    3. I rather agree
    4. No opinion
    5. I rather disagree
    6. I disagree very much
    7. I totally disagree
51. In most ways my life is close to my ideal.
    1. I totally agree
    2. I agree very much
    3. I rather agree
    4. No opinion
    5. I rather disagree
    6. I disagree very much
    7. I totally disagree
52. The conditions of my life are excellent.
    1. I totally agree
    2. I agree very much
    3. I rather agree
    4. No opinion
    5. I rather disagree
    6. I disagree very much
    7. I totally disagree
53. I am satisfied with my life.
    1. I totally agree
    2. I agree very much
    3. I rather agree
    4. No opinion
    5. I rather disagree
    6. I disagree very much
    7. I totally disagree
54. So far I have gotten the important things I want in life.
    1. I totally agree
    2. I agree very much
    3. I rather agree
    4. No opinion
    5. I rather disagree
    6. I disagree very much
    7. I totally disagree
55. If I could live my life over, I would change almost nothing.
    1. I totally agree
    2. I agree very much
    3. I rather agree
    4. No opinion
    5. I rather disagree
    6. I disagree very much
    7. I totally disagree

Age:

<20

20-29

30-39

40-49

# Questionnaire (native version: Polish language)

Część 1. Moje korzystanie z Instagramu

1. Jeżeli mogłabym odwiedzać tylko jedno miejsce w Internecie, byłby to Instagram.
   1. Całkowicie się zgadzam
   2. Bardzo się zgadzam
   3. Raczej się zgadzam
   4. Jest mi to obojętne
   5. Raczej się nie zgadzam
   6. Bardzo się nie zgadzam
   7. Całkowicie się nie zgadzam
2. Źle się czuję, jeżeli nie sprawdzę mojego profilu na Instagramie choć raz dziennie.
   1. Całkowicie się zgadzam
   2. Bardzo się zgadzam
   3. Raczej się zgadzam
   4. Jest mi to obojętne
   5. Raczej się nie zgadzam
   6. Bardzo się nie zgadzam
   7. Całkowicie się nie zgadzam
3. Często szukam dostępu do Internetu, by zajrzeć na Instagram.
   1. Całkowicie się zgadzam
   2. Bardzo się zgadzam
   3. Raczej się zgadzam
   4. Jest mi to obojętne
   5. Raczej się nie zgadzam
   6. Bardzo się nie zgadzam
   7. Całkowicie się nie zgadzam
4. Przed snem zazwyczaj zaglądam do Instagramu.
   1. Całkowicie się zgadzam
   2. Bardzo się zgadzam
   3. Raczej się zgadzam
   4. Jest mi to obojętne
   5. Raczej się nie zgadzam
   6. Bardzo się nie zgadzam
   7. Całkowicie się nie zgadzam
5. Przeglądanie Instagramu jest dobrym sposobem na nudę.
   1. Całkowicie się zgadzam
   2. Bardzo się zgadzam
   3. Raczej się zgadzam
   4. Jest mi to obojętne
   5. Raczej się nie zgadzam
   6. Bardzo się nie zgadzam
   7. Całkowicie się nie zgadzam
6. Kiedy się nudzę, korzystam czasem z Instagramu.
   1. Całkowicie się zgadzam
   2. Bardzo się zgadzam
   3. Raczej się zgadzam
   4. Jest mi to obojętne
   5. Raczej się nie zgadzam
   6. Bardzo się nie zgadzam
   7. Całkowicie się nie zgadzam
7. Kiedy się nudzę, zawsze korzystam z Instgramu.
   1. Całkowicie się zgadzam
   2. Bardzo się zgadzam
   3. Raczej się zgadzam
   4. Jest mi to obojętne
   5. Raczej się nie zgadzam
   6. Bardzo się nie zgadzam
   7. Całkowicie się nie zgadzam
8. Spędzam czas na Instagramie kosztem moich innych obowiązków.
   1. Całkowicie się zgadzam
   2. Bardzo się zgadzam
   3. Raczej się zgadzam
   4. Jest mi to obojętne
   5. Raczej się nie zgadzam
   6. Bardzo się nie zgadzam
   7. Całkowicie się nie zgadzam
9. Spędzam na Instagramie więcej czasu, niżbym chciała.
   1. Całkowicie się zgadzam
   2. Bardzo się zgadzam
   3. Raczej się zgadzam
   4. Jest mi to obojętne
   5. Raczej się nie zgadzam
   6. Bardzo się nie zgadzam
   7. Całkowicie się nie zgadzam
10. Zdarza się, że korzystam z Instagramu zamiast spać.
    1. Całkowicie się zgadzam
    2. Bardzo się zgadzam
    3. Raczej się zgadzam
    4. Jest mi to obojętne
    5. Raczej się nie zgadzam
    6. Bardzo się nie zgadzam
    7. Całkowicie się nie zgadzam
11. Mój profil na Instagramie jest dopracowany.
    1. Całkowicie się zgadzam
    2. Bardzo się zgadzam
    3. Raczej się zgadzam
    4. Jest mi to obojętne
    5. Raczej się nie zgadzam
    6. Bardzo się nie zgadzam
    7. Całkowicie się nie zgadzam
12. Lubię ulepszać mój profil na Instagramie.
    1. Całkowicie się zgadzam
    2. Bardzo się zgadzam
    3. Raczej się zgadzam
    4. Jest mi to obojętne
    5. Raczej się nie zgadzam
    6. Bardzo się nie zgadzam
    7. Całkowicie się nie zgadzam
13. To dla mnie ważne, by regularnie umieszczać nowe posty na Instagramie.
    1. Całkowicie się zgadzam
    2. Bardzo się zgadzam
    3. Raczej się zgadzam
    4. Jest mi to obojętne
    5. Raczej się nie zgadzam
    6. Bardzo się nie zgadzam
    7. Całkowicie się nie zgadzam
14. Kiedy porównuję swój profil na Instagramie do osób, które mają lepsze profile czuję, że jest możliwe, że pewnego dnia ja też osiągnę poziom ich popularności.
    1. Całkowicie się zgadzam
    2. Bardzo się zgadzam
    3. Raczej się zgadzam
    4. Jest mi to obojętne
    5. Raczej się nie zgadzam
    6. Bardzo się nie zgadzam
    7. Całkowicie się nie zgadzam
15. Kiedy porównuję swój profil na Instagramie do osób, które mają lepsze profile mam nadzieję, że mój profil się poprawi.
    1. Całkowicie się zgadzam
    2. Bardzo się zgadzam
    3. Raczej się zgadzam
    4. Jest mi to obojętne
    5. Raczej się nie zgadzam
    6. Bardzo się nie zgadzam
    7. Całkowicie się nie zgadzam

1. Kiedy porównuję swój profil na Instagramie do osób, które mają lepsze profile jestem zadowolona, że mój profil także może być tak dobry.
   1. Całkowicie się zgadzam
   2. Bardzo się zgadzam
   3. Raczej się zgadzam
   4. Jest mi to obojętne
   5. Raczej się nie zgadzam
   6. Bardzo się nie zgadzam
   7. Całkowicie się nie zgadzam
2. Kiedy porównuję swój profil na Instagramie do osób, które mają lepsze profile czuję się sfrustrowana poziomem mojego własnego profilu.
   1. Całkowicie się zgadzam
   2. Bardzo się zgadzam
   3. Raczej się zgadzam
   4. Jest mi to obojętne
   5. Raczej się nie zgadzam
   6. Bardzo się nie zgadzam
   7. Całkowicie się nie zgadzam
3. Kiedy porównuję swój profil na Instagramie do osób, które mają lepsze profile czuję niepokój, że mój profil nie osiąga takich wyników.
   1. Całkowicie się zgadzam
   2. Bardzo się zgadzam
   3. Raczej się zgadzam
   4. Jest mi to obojętne
   5. Raczej się nie zgadzam
   6. Bardzo się nie zgadzam
   7. Całkowicie się nie zgadzam
4. Kiedy porównuję swój profil na Instagramie do osób, które mają lepsze profile popadam w depresję uzmysławiając sobie, że mój profil nie jest tak dobry.
   1. Całkowicie się zgadzam
   2. Bardzo się zgadzam
   3. Raczej się zgadzam
   4. Jest mi to obojętne
   5. Raczej się nie zgadzam
   6. Bardzo się nie zgadzam
   7. Całkowicie się nie zgadzam
5. Kiedy porównuję swój profil na Instagramie do osób, które mają słabsze profile boję się, że mój profil się pogorszy.
   1. Całkowicie się zgadzam
   2. Bardzo się zgadzam
   3. Raczej się zgadzam
   4. Jest mi to obojętne
   5. Raczej się nie zgadzam
   6. Bardzo się nie zgadzam
   7. Całkowicie się nie zgadzam
6. Kiedy porównuję swój profil na Instagramie do osób, które mają słabsze profile boję się, że przyszłość mojego profilu będzie taka sama.
   1. Całkowicie się zgadzam
   2. Bardzo się zgadzam
   3. Raczej się zgadzam
   4. Jest mi to obojętne
   5. Raczej się nie zgadzam
   6. Bardzo się nie zgadzam
   7. Całkowicie się nie zgadzam
7. Kiedy porównuję swój profil na Instagramie do osób, które mają słabsze profile boję się, że popularność mojego profilu spadnie.
   1. Całkowicie się zgadzam
   2. Bardzo się zgadzam
   3. Raczej się zgadzam
   4. Jest mi to obojętne
   5. Raczej się nie zgadzam
   6. Bardzo się nie zgadzam
   7. Całkowicie się nie zgadzam
8. Kiedy porównuję swój profil na Instagramie do osób, które mają słabsze profile czuję, jak dobrze sobie radzę.
   1. Całkowicie się zgadzam
   2. Bardzo się zgadzam
   3. Raczej się zgadzam
   4. Jest mi to obojętne
   5. Raczej się nie zgadzam
   6. Bardzo się nie zgadzam
   7. Całkowicie się nie zgadzam
9. Kiedy porównuję swój profil na Instagramie do osób, które mają słabsze profile czuję ulgę odnośnie mojego własnego profilu.
   1. Całkowicie się zgadzam
   2. Bardzo się zgadzam
   3. Raczej się zgadzam
   4. Jest mi to obojętne
   5. Raczej się nie zgadzam
   6. Bardzo się nie zgadzam
   7. Całkowicie się nie zgadzam
10. Kiedy porównuję swój profil na Instagramie do osób, które mają słabsze profile jestem szczęśliwa, że tak dobrze sobie radzę.
    1. Całkowicie się zgadzam
    2. Bardzo się zgadzam
    3. Raczej się zgadzam
    4. Jest mi to obojętne
    5. Raczej się nie zgadzam
    6. Bardzo się nie zgadzam
    7. Całkowicie się nie zgadzam

Część 2. Moje samopoczucie.

1. W zasadzie jestem zadowolona z siebie samej.
   1. Całkowicie się zgadzam
   2. Bardzo się zgadzam
   3. Raczej się zgadzam
   4. Jest mi to obojętne
   5. Raczej się nie zgadzam
   6. Bardzo się nie zgadzam
   7. Całkowicie się nie zgadzam
2. Czasami myślę, że jestem do niczego.
   1. Całkowicie się zgadzam
   2. Bardzo się zgadzam
   3. Raczej się zgadzam
   4. Jest mi to obojętne
   5. Raczej się nie zgadzam
   6. Bardzo się nie zgadzam
   7. Całkowicie się nie zgadzam
3. Uważam, że posiadam kilka dobrych cech.
   1. Całkowicie się zgadzam
   2. Bardzo się zgadzam
   3. Raczej się zgadzam
   4. Jest mi to obojętne
   5. Raczej się nie zgadzam
   6. Bardzo się nie zgadzam
   7. Całkowicie się nie zgadzam
4. Mogę robić rzeczy tak samo dobrze jak inni.
   1. Całkowicie się zgadzam
   2. Bardzo się zgadzam
   3. Raczej się zgadzam
   4. Jest mi to obojętne
   5. Raczej się nie zgadzam
   6. Bardzo się nie zgadzam
   7. Całkowicie się nie zgadzam
5. Czuję, że nie mam zbytnich powodów do dumy.
   1. Całkowicie się zgadzam
   2. Bardzo się zgadzam
   3. Raczej się zgadzam
   4. Jest mi to obojętne
   5. Raczej się nie zgadzam
   6. Bardzo się nie zgadzam
   7. Całkowicie się nie zgadzam
6. Czasami czuję się bezużyteczna.
   1. Całkowicie się zgadzam
   2. Bardzo się zgadzam
   3. Raczej się zgadzam
   4. Jest mi to obojętne
   5. Raczej się nie zgadzam
   6. Bardzo się nie zgadzam
   7. Całkowicie się nie zgadzam

1. Uważam, że jestem wartościową osobą, przynajmniej tak samo jak reszta.
   1. Całkowicie się zgadzam
   2. Bardzo się zgadzam
   3. Raczej się zgadzam
   4. Jest mi to obojętne
   5. Raczej się nie zgadzam
   6. Bardzo się nie zgadzam
   7. Całkowicie się nie zgadzam
2. Chciałabym mieć dla siebie więcej szacunku.
   1. Całkowicie się zgadzam
   2. Bardzo się zgadzam
   3. Raczej się zgadzam
   4. Jest mi to obojętne
   5. Raczej się nie zgadzam
   6. Bardzo się nie zgadzam
   7. Całkowicie się nie zgadzam
3. W sumie to czuję, że jestem porażką.
   1. Całkowicie się zgadzam
   2. Bardzo się zgadzam
   3. Raczej się zgadzam
   4. Jest mi to obojętne
   5. Raczej się nie zgadzam
   6. Bardzo się nie zgadzam
   7. Całkowicie się nie zgadzam
4. Mam do siebie pozytywne nastawienie.
   1. Całkowicie się zgadzam
   2. Bardzo się zgadzam
   3. Raczej się zgadzam
   4. Jest mi to obojętne
   5. Raczej się nie zgadzam
   6. Bardzo się nie zgadzam
   7. Całkowicie się nie zgadzam
5. Czuję napięcie lub zdenerwowanie.
   1. Całkowicie się zgadzam
   2. Bardzo się zgadzam
   3. Raczej się zgadzam
   4. Jest mi to obojętne
   5. Raczej się nie zgadzam
   6. Bardzo się nie zgadzam
   7. Całkowicie się nie zgadzam
6. Wciąż potrafię się cieszyć rzeczami, którymi zwykłam się cieszyć.
   1. Całkowicie się zgadzam
   2. Bardzo się zgadzam
   3. Raczej się zgadzam
   4. Jest mi to obojętne
   5. Raczej się nie zgadzam
   6. Bardzo się nie zgadzam
   7. Całkowicie się nie zgadzam
7. Mam przeczucie, że wydarzy się coś złego.
   1. Całkowicie się zgadzam
   2. Bardzo się zgadzam
   3. Raczej się zgadzam
   4. Jest mi to obojętne
   5. Raczej się nie zgadzam
   6. Bardzo się nie zgadzam
   7. Całkowicie się nie zgadzam
8. Potrafię się śmiać i dostrzegać zabawne strony każdej sytuacji.
   1. Całkowicie się zgadzam
   2. Bardzo się zgadzam
   3. Raczej się zgadzam
   4. Jest mi to obojętne
   5. Raczej się nie zgadzam
   6. Bardzo się nie zgadzam
   7. Całkowicie się nie zgadzam
9. Zamartwiam się.
   1. Całkowicie się zgadzam
   2. Bardzo się zgadzam
   3. Raczej się zgadzam
   4. Jest mi to obojętne
   5. Raczej się nie zgadzam
   6. Bardzo się nie zgadzam
   7. Całkowicie się nie zgadzam
10. Czuję się radosna.
    1. Całkowicie się zgadzam
    2. Bardzo się zgadzam
    3. Raczej się zgadzam
    4. Jest mi to obojętne
    5. Raczej się nie zgadzam
    6. Bardzo się nie zgadzam
    7. Całkowicie się nie zgadzam
11. Potrafię spokojnie usiąść i poczuć się zrelaksowana.
    1. Całkowicie się zgadzam
    2. Bardzo się zgadzam
    3. Raczej się zgadzam
    4. Jest mi to obojętne
    5. Raczej się nie zgadzam
    6. Bardzo się nie zgadzam
    7. Całkowicie się nie zgadzam
12. Czuję, że funkcjonuję na zwolnionych obrotach.
    1. Całkowicie się zgadzam
    2. Bardzo się zgadzam
    3. Raczej się zgadzam
    4. Jest mi to obojętne
    5. Raczej się nie zgadzam
    6. Bardzo się nie zgadzam
    7. Całkowicie się nie zgadzam
13. Czuję strach, „motyle w żołądku”.
    1. Całkowicie się zgadzam
    2. Bardzo się zgadzam
    3. Raczej się zgadzam
    4. Jest mi to obojętne
    5. Raczej się nie zgadzam
    6. Bardzo się nie zgadzam
    7. Całkowicie się nie zgadzam
14. Przestałam dbać o swój wygląd.
    1. Całkowicie się zgadzam
    2. Bardzo się zgadzam
    3. Raczej się zgadzam
    4. Jest mi to obojętne
    5. Raczej się nie zgadzam
    6. Bardzo się nie zgadzam
    7. Całkowicie się nie zgadzam
15. Czuję się niespokojna gdy mam coś zrobić.
    1. Całkowicie się zgadzam
    2. Bardzo się zgadzam
    3. Raczej się zgadzam
    4. Jest mi to obojętne
    5. Raczej się nie zgadzam
    6. Bardzo się nie zgadzam
    7. Całkowicie się nie zgadzam
16. Patrzę z radością w przyszłość.
    1. Całkowicie się zgadzam
    2. Bardzo się zgadzam
    3. Raczej się zgadzam
    4. Jest mi to obojętne
    5. Raczej się nie zgadzam
    6. Bardzo się nie zgadzam
    7. Całkowicie się nie zgadzam
17. Zdarzają mi się napady paniki.
    1. Całkowicie się zgadzam
    2. Bardzo się zgadzam
    3. Raczej się zgadzam
    4. Jest mi to obojętne
    5. Raczej się nie zgadzam
    6. Bardzo się nie zgadzam
    7. Całkowicie się nie zgadzam
18. Potrafię czerpać radość z dobrej książki, słuchowiska czy filmu.
    1. Całkowicie się zgadzam
    2. Bardzo się zgadzam
    3. Raczej się zgadzam
    4. Jest mi to obojętne
    5. Raczej się nie zgadzam
    6. Bardzo się nie zgadzam
    7. Całkowicie się nie zgadzam
19. Uważam, że moje życie jest bliskie ideału.
    1. Całkowicie się zgadzam
    2. Bardzo się zgadzam
    3. Raczej się zgadzam
    4. Jest mi to obojętne
    5. Raczej się nie zgadzam
    6. Bardzo się nie zgadzam
    7. Całkowicie się nie zgadzam
20. Warunki mojego życia są wspaniałe
    1. Całkowicie się zgadzam
    2. Bardzo się zgadzam
    3. Raczej się zgadzam
    4. Jest mi to obojętne
    5. Raczej się nie zgadzam
    6. Bardzo się nie zgadzam
    7. Całkowicie się nie zgadzam

1. Jestem usatysfakcjonowana moim życiem.
   1. Całkowicie się zgadzam
   2. Bardzo się zgadzam
   3. Raczej się zgadzam
   4. Jest mi to obojętne
   5. Raczej się nie zgadzam
   6. Bardzo się nie zgadzam
   7. Całkowicie się nie zgadzam
2. Jak dotąd osiągam w życiu rzeczy, które uważam za ważne.
   1. Całkowicie się zgadzam
   2. Bardzo się zgadzam
   3. Raczej się zgadzam
   4. Jest mi to obojętne
   5. Raczej się nie zgadzam
   6. Bardzo się nie zgadzam
   7. Całkowicie się nie zgadzam
3. Gdybym mogła przeżyć moje życie na nowo, nic bym nie zmieniła.
   1. Całkowicie się zgadzam
   2. Bardzo się zgadzam
   3. Raczej się zgadzam
   4. Jest mi to obojętne
   5. Raczej się nie zgadzam
   6. Bardzo się nie zgadzam
   7. Całkowicie się nie zgadzam

**Metryczka**

Wiek

<20

20-29

30-39

40-49
